# Statistical Inference of Phenotype-Specific Molecular Mechanisms from Cell Line-Specific Gene Regulatory Networks with Application to Quizartinib Sensitivity

**DOI:** 10.3390/ijms27093885

**Published:** 2026-04-27

**Authors:** Jooee Oh, Heewon Park

**Affiliations:** 1 Department of Statistics, Sungshin Women’s University, Seoul 02844, Republic of Korea; 2 School of Mathematics Statistics and Data Science, Sungshin Women’s University, Seoul 01133, Republic of Korea; 3 Data Science Center, Sungshin Women’s University, Seoul 02844, Republic of Korea; 4 M&D Data Science Center, Institute of Science Tokyo, Tokyo 113-8510, Japan; 5 Human Genome Center, Institute of Medical Science, University of Tokyo, 4-6-1 Shirokane-dai, Minato-ku, Tokyo 108-8639, Japan

**Keywords:** cell line-specific analysis, gene regulatory network, differentially regulated gene network, acute myeloid leukemia, Quizartinib

## Abstract

Gene regulatory networks differ substantially across individual cell lines, and population-level network inferences often fail to capture the underlying biological heterogeneity. To better capture this heterogeneity, cell line-specific gene network analysis is required. However, interpreting such high-dimensional cell line-specific networks remains a major challenge in the field of network biology. One interpretative approach is to identify differentially regulated gene networks (DGNs) between phenotypes because these networks can highlight phenotype-specific regulatory mechanisms. Although several methods have been proposed for DGN analysis, they are not suitable for cell line-specific gene network analysis, which is characterized by pronounced heterogeneity across individual networks. To address this problem, we proposed a novel statistical method for identifying DGNs in a cell line-specific scenario. The proposed framework integrates cell line-specific network estimation, a Kullback–Leibler divergence-based comparison of multivariate distributions, and a DKL-ratio statistic to quantify between-phenotype heterogeneity relative to within-phenotype homogeneity. Our method evaluates both between-phenotype heterogeneity and within-phenotype homogeneity, ensuring the robust detection of phenotype-specific network structures. Through Monte Carlo simulation studies, we systematically evaluated the performance of the proposed method and demonstrated that our strategy consistently outperformed existing methods in terms of accuracy, precision, true positive rate (TPR), true negative rate (TNR), and F-measure across diverse network structures and mean shift scenarios. Statistical significance was assessed using a permutation-based framework, confirming that the identified networks are unlikely to arise from random variation. We further applied our strategy to Quizartinib sensitivity-specific gene network analysis and identified immune-related subnetworks enriched in antigen processing and presentation pathways. These subnetworks included hub genes such as IFIT1, PSMB9, and HLA-B, which are known to be associated with immune evasion and drug resistance in acute myeloid leukemia. Our findings demonstrate that the proposed method enables statistically reliable and biologically interpretable identification of phenotype-specific gene regulatory mechanisms, providing insights into potential therapeutic targets.

## 1. Introduction

Gene network analysis is crucial for understanding disease mechanisms, which are often driven by intricate interactions among multiple genes. To estimate gene networks effectively, various computational strategies have been developed and applied to uncover the complex mechanisms of diseases [1,2,3]. However, most network inference techniques construct a single population-level gene network that fails to capture the cell-line-specific regulatory structures of individual cell lines. Each cell line has its own unique gene network because molecular interplay varies across individual cell lines. Therefore, a single aggregate network cannot accurately describe the cell line-specific molecular interplay and may overlook meaningful biological variability. To capture this heterogeneity, we focused on cell line-specific gene network analysis.

Since the estimated network is usually represented in matrix form containing more than tens of thousands of genes, the interpretation of such a large and complex structure remains a significant challenge. One effective strategy to address this issue is to identify and analyze differentially regulated gene networks (DGNs), which uncover distinct regulatory patterns between different phenotypes (e.g., normal and cancer, anti-cancer drug-sensitive and-resistant cell lines). To this end, several computational approaches have been developed to detect DGNs by comparing gene networks inferred from the same system under different states, such as healthy vs. diseased tissues, or under genetic perturbations (e.g., knockouts) [4,5,6]. These methods focus on differences in gene–gene interactions, providing deeper insight into phenotype-specific molecular mechanisms than traditional expression analyses.

However, most existing DGN identification methods are based on population-level analyses, where a single representative network is estimated for each phenotype by aggregating samples, thereby implicitly assuming homogeneity within each group [7]. Thus, these approaches may not adequately capture variability within phenotypes, limiting their applicability to cell line-specific frameworks. To overcome this limitation, we proposed a statistical framework, termed CellDGN, for identifying DGNs between phenotypes in a cell line-specific context. In this study, we formulate the following hypothesis testing: The null hypothesis ($H_{0}$) posits that all cell lines possess a random network structure, implying that molecular interactions are distributed stochastically without specific regulatory patterns. The alternative hypothesis ($H_{1}$) is the negation of $H_{0}$, suggesting the existence of non-random, cell-line-specific regulatory architectures. We define population-level approaches as methods that estimate a single representative network for each phenotype by aggregating samples within the group, thereby implicitly assuming homogeneity among samples. While numerous statistical methods have been developed to investigate heterogeneity within cellular and organismal phenotypes, most existing DGN identification approaches do not explicitly model network variability at the level of individual cell lines. This limitation motivates the need for methods that can capture cell line-specific network heterogeneity. A truly phenotype-specific network should fulfill two criteria: (1) it should differ across phenotypes (between-phenotype heterogeneity) and (2) it should consistently recur within the same phenotype (within-phenotype homogeneity). Building on these criteria, our method is designed to simultaneously capture differences across phenotypes while ensuring consistency within each phenotype, enabling robust identification of phenotype-specific network structures at the cell line level. Our strategy explicitly evaluates both conditions, enabling the robust identification of phenotype-specific molecular interplays from cell line-specific gene networks.

We demonstrate the performance of our method through Monte Carlo simulations, showing that the proposed approach achieves superior performance compared to existing DGN identification methods in detecting phenotype-specific network structures. We further applied our method to the Genomics of Drug Sensitivity in Cancer (GDSC) dataset, focusing on cell lines resistant to Quizartinib, a Food and Drug Administration (FDA)-approved drug for acute myeloid leukemia (AML). Our analysis successfully uncovered the DGNs associated with drug resistance. Notably, these networks were enriched in immune-related genes, particularly those involved in MHC class I-mediated antigen presentation, highlighting their potential role in immune evasion and drug resistance. Hub genes within these subnetworks demonstrated strong biological relevance, suggesting new possibilities for the development of therapies. Collectively, these results substantiate the ability of our strategy to accurately capture differentially regulated networks within a cell line-specific framework. Beyond the traditional DGN analysis, our approach emphasizes the importance of jointly accounting for between-phenotype heterogeneity and within-phenotype consistency to achieve robust and biologically meaningful network inference.

## 2. Results

The results are presented in two parts: Monte Carlo simulation studies and the analysis of AML drug sensitivity-specific gene networks. First, Monte Carlo simulations are conducted to evaluate the performance of the proposed method. Next, Quizartinib resistance-specific gene networks were identified using the proposed method and their biological relevance was examined.

### 2.1. Monte Carlo Simulation Studies

**Figure 1:**
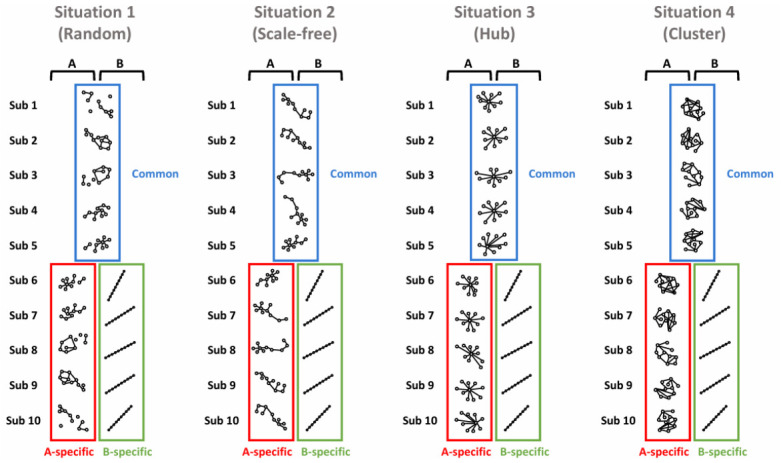
Network topologies for four simulation scenarios representing different patterns of between-phenotype heterogeneity and within-phenotype homogeneity. The networks in blue, red, and green boxes denote common subnetworks and phenotype A- and B-specific subnetworks, respectively. Each situation illustrates a distinct network topology (e.g., random, scale-free, hub-based, and clustered structures) used to evaluate the performance of the proposed method under controlled settings.

We considered a two-phenotype setting (A and B), and focused on phenotype A. We evaluated whether the proposed method could accurately identify A-specific gene networks while avoiding the detection of non–A-specific networks. As shown in Figure 1, we consider four network topologies: random, scale-free, hub, and cluster. Each cell line-specific gene network consisted of 10 subnetworks. Among these 10, subnetworks 1–5 were common to both phenotypes, whereas subnetworks 6–10 were phenotype-specific subnetworks. The total number of genes was fixed at p=100 such that each of the 10 subnetworks contained 10 genes.

To simulate subnetworks with predefined structures, we generated a baseline precision matrix $\Omega \in R^{10\times 10}$ for each subnetwork using the huge.generator function in the R package huge. Notably, Ω was not estimated from real biological data, but randomly generated to provide synthetic network topologies (e.g., hub or scale-free structures) under a controlled simulation setting. Specifically, we created each five precision matrices for the A-specific (i.e., $\Omega_{A}^{\left(1\right)},\cdots ,\Omega_{A}^{\left(5\right)}$), B-specific (i.e., $\Omega_{B}^{\left(1\right)},\cdots ,\Omega_{B}^{\left(5\right)}$) and the common (i.e., $\Omega_{C}^{\left(1\right)},\cdots ,\Omega_{C}^{\left(5\right)}$) subnetworks, respectively. Common and A-specific subnetworks were allowed to adopt any of the four network structures (random, scale-free, hub, and cluster), whereas B-specific subnetworks were always generated with only a banded topology. To further ensure that the identification of DGNs is not biased by broad topological differences, we conducted an additional simulation where both phenotypes A and B share a banded topology. For the phenotype-specific subnetworks 6–10, we first generated a base precision matrix $\Omega_{A}$ for the sensitive group using a banded structure. The base precision matrix for the resistant group, $\Omega_{B}$, was then constructed by randomly removing 50% of the existing edges from the adjacency structure of $\Omega_{A}$. The total number of cell lines was n=120, indexed from 1 to 120. Among these, cell lines 1–40 (i.e., $n_{A}=40$) and 81–120 (i.e., $n_{B}=40$) were assigned to phenotypes A and B, respectively, while the remaining cell lines (41–80) were treated as intermediate (moderate) cell lines and not assigned to either phenotype. Each cell line has a phenotypic value, referred to as a modulator value, which is obtained by generating *n* cell lines from uniform distribution U(0,3) and sorting them in increasing order, resulting in $m_{1}<m_{2}<\cdots <m_{n}$. This value represents a cell-line-specific characteristic and induces heterogeneity within each phenotype. Based on this modulator, we generated a cell line-specific precision matrix $\Omega_{\alpha }$ by scaling the baseline precision matrix Ω (i.e., $\Omega_{A},\Omega_{B}$ and $\Omega_{C}$ for phenotype A, B -specific and common precision matrices, respectively) with a cell line-specific factor $v_{\alpha }$, which was randomly drawn from a uniform distribution and then ordered according to the modulator values as follows:

(1) $$\Omega_{\alpha }=v_{\alpha }\Omega ,\;v_{\alpha }∼U\left(0,3\right),\;v_{\alpha }\propto m,\;\alpha =1,\ldots ,n.$$

where $v_{\alpha }$ serves as a modeling device to mimic realistic cell line-to-cell line variability in gene regulatory association strengths rather than representing a specific biological parameter. This formulation preserves a common underlying network topology (i.e., the same sparsity pattern of edges) across all cell lines, while allowing heterogeneous interaction strengths through varying edge weights.

We also evaluated whether our method works well when the two phenotypes differ not only in network structure but also at the expression level. Three phenotype-wise mean scenarios are considered. In the first scenario, both groups share the same mean of expression levels of genes (i.e., $\mu_{A}=0$ and $\mu_{B}=0$), indicating no mean shift. In the second scenario, there is a mild shift in the B phenotype (i.e., $\mu_{A}=0$ and $\mu_{B}=1$), whereas the third scenario modeled a strong shift (i.e., $\mu_{A}=0$ and $\mu_{B}=5$).

For each cell line, gene expression values were generated from a multivariate normal distribution, the covariance of which was computed as the inverse of the precision matrix defined by the corresponding phenotype and subnetwork-specific precision matrix. For phenotype A, the expression level vectors in subnetworks 1–5 (i.e., nw = 1, …, 5), which are shared by both phenotypes, were generated as follows:

(2) $$x_{\alpha }^{\left(\mathrm{nw}\right)}∼N_{p}\left(0,{\left(\Omega_{\alpha ,C}^{\left(\mathrm{nw}\right)}\right)}^{-1}\right),\;\mathrm{nw}=1,\cdots ,5,$$

where $N_{p}\left(\cdot \right)$ denotes a *p*-dimensional multivariate normal distribution and *p* represents the number of genes (i.e., the dimension of the expression vector) in each subnetwork. For subnetworks 6–10 (i.e., nw = 6, …, 10), which are specific to phenotype A, we generated the expression levels of the genes as follows:

(3) $$x_{\alpha }^{\left(\mathrm{nw}\right)}∼N_{p}\left(\mu_{A},{\left(\Omega_{\alpha ,A}^{\left(\mathrm{nw}\right)}\right)}^{-1}\right),\;\mathrm{nw}=6,\cdots ,10.$$

For phenotype B, we used the same logic, that is, subnetworks 1–5 from the common precision matrices and subnetworks 6–10 from the B-specific (i.e., banded) precision matrices.

Using the generated gene expression levels, we estimated cell line-specific gene networks using a kernel–based $L_{1}$-type regularization method [8]. For the αth cell line and *ℓ*th target gene, we estimated the coefficient vector ${\hat{\beta }}_{\ell \alpha }\in R^{p-1}$ and variance ${\hat{\sigma }}_{\ell \alpha }^{2}$. We then computed the cell line-specific precision matrix ${\hat{\Omega }}_{\alpha }$ according to Meinshausen and Bühlmann [9]:

(4) $${\left({\hat{\Omega }}_{\alpha }\right)}_{\ell ,\ell }=\frac{1}{{\hat{\sigma }}_{\ell \alpha }^{2}}\;\mathrm{and}\;{\left({\hat{\Omega }}_{\alpha }\right)}_{\ell ,-\ell }=-{\left({\hat{\Omega }}_{\alpha }\right)}_{\ell ,\ell }{\hat{\beta }}_{\ell \alpha }^{⊤},$$

where ${\left({\hat{\Omega }}_{\alpha }\right)}_{\ell ,\ell }$ denotes the *ℓ*th diagonal element of ${\hat{\Omega }}_{\alpha }$ and ${\left({\hat{\Omega }}_{\alpha }\right)}_{\ell ,-\ell }$ denotes the *ℓ*th row of ${\hat{\Omega }}_{\alpha }$ with *ℓ*th element removed. A detailed derivation of the derivative of Ω, leading to the estimation of the precision matrix, is provided in Appendix A. Since the estimated edge weights were not symmetric, we applied a transformation to obtain the symmetric precision matrix as follows:

(5) $${\hat{\Omega }}_{\alpha }\leftarrow \frac{{\hat{\Omega }}_{\alpha }+{\hat{\Omega }}_{\alpha }^{⊤}}{2}\;\mathrm{and}\;{\hat{\Sigma }}_{\alpha }={\hat{\Omega }}_{\alpha }^{-1}.$$

The Kullback–Leibler (KL) divergence between networks of the αth and *i*th cell lines was evaluated based on ${\hat{\Sigma }}_{\alpha }$ in (5) as follows,

(6) $$D_{\mathrm{KL}}=\frac{1}{2}\left[\log\left\{\frac{|{\hat{\Sigma }}_{i}|}{|{\hat{\Sigma }}_{\alpha }|}\right\}-p+\mathrm{tr}\left\{{\hat{\Sigma }}_{i}^{-1}{\hat{\Sigma }}_{\alpha }\right\}+{\left(\mu_{\alpha }-\mu_{i}\right)}^{⊤}{\hat{\Sigma }}_{i}^{-1}\left(\mu_{\alpha }-\mu_{i}\right)\right],$$

and DGNs were identified using our strategy. The performance of the proposed method was evaluated in terms of the accuracy, precision, true positive rate (TPR), true negative rate (TNR), and F-measure. We then compared our method with existing approaches: DGNdetector [7], SAM-GS [10], GSCA [11], and DiffNetFDR [12]. All methods aim to identify DGNs, but differ in the information they utilize:

- Proposed: (${\hat{\mu }}_{\alpha \in A},{\hat{\Sigma }}_{\alpha \in A}$) ↔ (${\hat{\mu }}_{\alpha \in B},{\hat{\Sigma }}_{\alpha \in B}$)
- DGNdetector: (${\hat{\mu }}_{A},{\hat{\Sigma }}_{A}$) ↔ (${\hat{\mu }}_{B},{\hat{\Sigma }}_{B}$)
- SAM-GS: (${\hat{\mu }}_{A}$) ↔ (${\hat{\mu }}_{B}$)
- GSCA: (${\hat{\Sigma }}_{A}$) ↔ (${\hat{\Sigma }}_{B}$)

Table 1 summarizes the results of the DGN identification in the cell line-specific framework. As shown in Table 1, the methods that account for both the mean differences and network topology (i.e., the proposed framework and DGNdetector) provide effective results overall. In particular, the proposed method consistently outperformed all competing approaches across all scenarios, showing stable accuracy regardless of the degree of the mean difference or network structure complexity. Furthermore, even in cases where the networks shared the same banded topology structure, the proposed methodology demonstrated superior performance in identifying phenotype-associated rewiring compared to other methods. In contrast, SAM-GS performed well for scenarios with large differences in means, but its performance degraded notably when the mean shift was small. The GSCA cannot perform well overall.

We also considered simulations with a larger-scale network comprising 200 genes, where each subnetwork contained 20 genes, as listed in Table 2. As the number of genes increased, the competing approaches exhibited severe performance degradation, whereas the proposed method exhibited a clear improvement in performance. Across all evaluation metrics (accuracy, precision, TPR, TNR, and F-measure), the proposed method consistently achieved outstanding results. These results suggest that our approach is well-suited for large-scale gene network analysis.

This performance gap may be attributed to the limited use of information in the competing methods, which may not fully capture the complex structure of cell line-specific gene networks. In contrast, the superior performance of the proposed method can be attributed to its integrative use of multiple sources of information, enabling a more comprehensive characterization of cell line-specific gene network differences.

### 2.2. AML Drug Sensitivity-Specific Gene Network Analysis

We applied our methodology to the Genomics of Drug Sensitivity in Cancer (GDSC) database (www.cancerrxgene.org), which provides gene expression profiles and drug response information for diverse cancer cell lines. Specifically, the preprocessed basal gene expression dataset (Cell_line_RMA_proc_basalExp.txt) and the drug response dataset (GDSC1_fitted_dose_response_27Oct23.xlsx) were downloaded from the GDSC data portal. The GDSC1 release represents an earlier large-scale pharmacogenomic screening dataset, and the file name indicates the version updated on 27 October 2023. The dataset included gene expression profiles for 1020 cell lines across 9765 genes and drug sensitivity data for 970 cell lines across 402 drugs, quantified using IC50 values (i.e., the drug concentration required to inhibit cell growth by 50%; lower IC50 indicates higher sensitivity). Among these drugs, we focused on Quizartinib, an FDA-approved drug for acute myeloid leukemia (AML), to explore drug sensitivity-specific gene networks.

We estimated cell line-specific gene networks for 20 cell lines, including 10 Quizartinib-sensitive cell lines (lowest IC50) and 10 Quizartinib-resistant cell lines (highest IC50). In total, 20 individual gene networks were constructed, one for each cell line, yielding 20 corresponding precision matrices that reflect cell line-specific network structures for both sensitive and resistant groups.

We estimated cell line-specific gene regulatory networks for a total of 20 cell lines selected based on their response to Quizartinib, including 10 sensitive cell lines with the lowest IC50 values and 10 resistant cell lines with the highest IC50 values. For each cell line, an individual gene network was constructed, resulting in 20 corresponding precision matrices that capture cell line-specific network structures across both sensitive and resistant groups.

**Figure 2:**
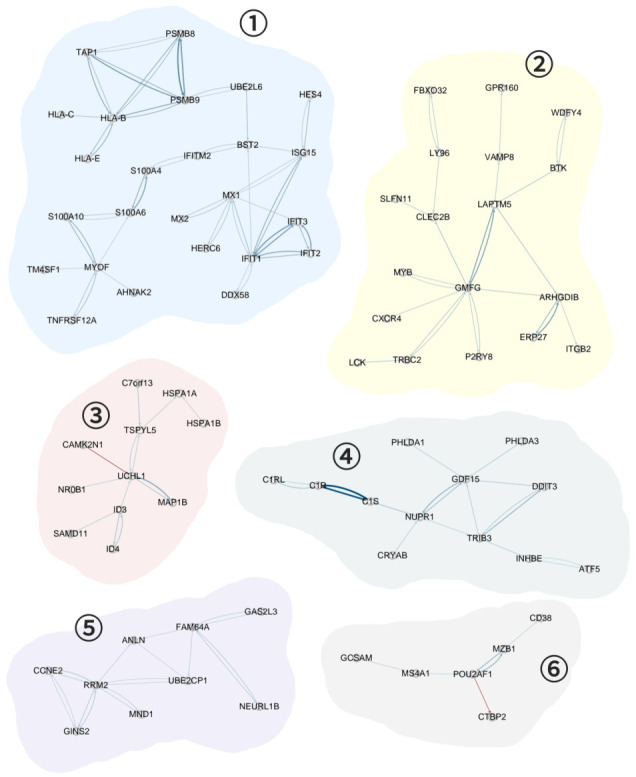
Differentially regulated gene networks in the most Quizartinib-resistant cell line. The edge color indicates the sign of the weight (red for negative, blue for positive). The thickness and intensity of edge indicates the strength of edge; darker and thicker edge corresponds to stronger interaction. The six highlighted subnetworks represent statistically significant differential subnetworks identified from the estimated full network, each capturing regions with pronounced differences in network structure between phenotypes.

For network construction, we selected the 1000 most variable genes with the highest variance across cell lines and employed nodewise regression based on neighborhood selection [8]. Specifically, we fitted 1000 regression models, each modeling one gene as the response (i.e., target gene) and all the remaining genes as predictors (i.e., regulator genes). This allowed us to estimate Quizartinib sensitivity-specific gene networks. Our primary focus was on the gene network of the most resistant cell line. To identify meaningful modules, we applied a threshold corresponding to the top 0.5% of absolute edge weights, removing all edges with weights below this cutoff. This filtering step resulted in a network consisting of 286 nodes and 437 edges. The thresholded network was then decomposed into subnetworks by identifying its connected components. Specifically, each subnetwork was defined as a set of genes connected through the retained edges. Using this definition, the filtered network was partitioned into 61 connected components, which were subsequently analyzed using our proposed strategy. We then applied our proposed strategy to the 61 subnetworks and identified 25 Quizartinib-resistant-specific gene networks at a significance level of α<0.05. The major Quizartinib-resistant-specific gene networks that contain at least five genes are illustrated in Figure 2.

Shown in Figure 2, the identified subnetworks consist of sparsely connected but structured groups of genes, where nodes represent genes and edges indicate inferred interactions. The identified subnetworks are derived from the application of a stringent threshold based on the top 0.5% of absolute edge weights, resulting in the retention of only the strongest associations. In the identified networks, positive interactions (blue edges) appear to be more prominent. This may be because positive edges tend to have relatively larger absolute edge weights than negative ones, and thus are more likely to be retained under the applied threshold. Overall, the resulting subnetworks highlight clusters of genes with strong positive associations, suggesting coordinated molecular interplays within Quizartinib-resistant-specific gene networks.

To support the biological relevance and reliability of our method, we summarized hub genes to a high degree in these major subnetworks and provided supporting evidence from the literature in Table 3. Below, we describe the biological characteristics of subnetworks in detail.

- **Subnetwork 1**
  Subnetwork 1 contains hub genes including *IFIT1*, *PSMB9*, and *HLA-B*, which have been previously reported to be associated with immune evasion or immunosuppression in AML. *IFIT1* is an interferon-stimulated gene upregulated in AML. It modulates immune responses, and its overexpression is linked to impaired apoptosis and poor prognosis, suggesting a role in immune evasion [13]. *PSMB9*, a core immunoproteasome component, is essential for MHC class I antigen presentation. Its repression in AML impairs immune recognition, while restoring its expression can reactivate cytotoxic T cell responses [14]. *HLA-B* is a classical MHC class I molecule presenting intracellular peptides to T and NK cells. Its expression level influences immune activity and clinical outcomes, with higher expression enhancing NK cell-mediated cytotoxicity [15]. Collectively, these findings suggest that Subnetwork 1 may represent an immune evasion module that contributes to Quizartinib resistance through impaired antigen presentation and reduced cytotoxic immune activity. This interpretation is supported by previous studies showing that loss or downregulation of MHC class I antigen presentation enables cancer cells to evade immune surveillance by impairing recognition by cytotoxic T lymphocytes, thereby weakening anti-tumor immune responses [16].
- **Subnetwork 2**
  Subnetwork 2 includes hub genes *GMFG*, *LAPTM5*, and *ARHGDIB*, all of which have been previously implicated in immune-related functions or leukemic pathogenesis in AML. *GMFG* regulates actin cytoskeleton remodeling and immune cell migration. In AML, elevated *GMFG* expression correlates with poor overall survival and increased leukemic cell motility, suggesting its role in disease progression and immune evasion [17]. *LAPTM5* is predominantly expressed in hematopoietic cells and participates in lysosome-mediated signaling. Its dysregulation in AML is associated with impaired immune regulation and altered myeloid differentiation [18]. *ARHGDIB*, encoding Rho GDP-dissociation inhibitor beta, has been identified as the source of a leukemia-associated minor histocompatibility antigen, LB-ARHGDIB-1R. This antigen elicits cytotoxic T cell responses against AML cells without inducing graft-versus-host disease, highlighting its potential for immunotherapy [19]. These interactions indicate that Subnetwork 2 may be involved in regulating cytoskeletal dynamics and immune cell signaling, potentially contributing to leukemic progression and drug resistance. This interpretation is supported by prior studies showing that cytoskeletal regulatory proteins are critical drivers of leukemia progression [20], and that actin cytoskeleton remodeling can suppress immune cell-mediated cytotoxicity, thereby facilitating immune evasion and therapeutic resistance [21].
- **Subnetwork 3**
  Subnetwork 3 contains hub genes including *GDF15* and *TRIB3*, which have been previously reported to be involved in leukemic cell survival and resistance-related pathways in AML. *GDF15* is a stress-responsive cytokine that plays roles in immune regulation and tumor progression. In AML, it is frequently overexpressed and contributes to an immunosuppressive microenvironment and treatment resistance, highlighting its relevance as a biomarker for disease severity and drug response [22,23]. *TRIB3* acts as a modulator of cell signaling pathways involved in proliferation and survival. Its dysregulation in AML has been linked to enhanced leukemic growth and adverse clinical outcomes, indicating its potential function in leukemogenesis and therapy resistance [24]. Our results suggest that Subnetwork 3 may be associated with transcriptional regulation and epigenetic modulation, which are known to influence leukemic cell plasticity and drug response. Dysregulation of transcriptional and epigenetic programs can alter gene expression patterns critical for maintaining leukemic cell survival and adaptation under therapeutic pressure, thereby contributing to drug resistance [25,26].
- **Subnetwork 4**
  Subnetwork 4 includes hub genes such as *UCHL1*, *TSPYL5*, and *ID3*, all of which are implicated in AML progression through diverse mechanisms. *UCHL1* is a deubiquitinating enzyme that modulates protein degradation and has been identified as an oncogene in AML. Its overexpression promotes leukemic cell proliferation and survival, making it a potential therapeutic target [27]. *TSPYL5* acts as a chromatin modifier and has been shown to promote cell cycle progression and suppress p53-mediated apoptosis in AML cells, thereby facilitating leukemogenesis [28]. *ID3* is a transcriptional regulator involved in cell differentiation. Its downregulation in AML correlates with poor prognosis, increased blast counts, and unfavorable genetic mutations such as FLT3 and NPM1, suggesting its role as a tumor suppressor and a prognostic biomarker [29]. These interactions indicate that Subnetwork 4 may be involved in apoptosis regulation and cell survival signaling pathways. Disruption of apoptotic signaling is a well-established mechanism of cancer drug resistance, allowing leukemic cells to evade therapy-induced cell death and maintain survival despite treatment [30].
- **Subnetwork 5**
  Subnetwork 5 includes hub genes *RRM2* and *FAM64A*, both of which contribute to leukemic cell proliferation and are associated with poor prognosis in AML. *RRM2* is essential for DNA synthesis and repair by regulating deoxyribonucleotide production. In AML, *RRM2* is frequently overexpressed, which promotes leukemic cell proliferation and confers resistance to chemotherapy. Its elevated expression correlates with adverse clinical outcomes and makes it a potential therapeutic target [31]. *FAM64A*, also known as PIMREG, is a cell cycle-related gene that enhances proliferation in AML. High expression of *FAM64A* has been linked to increased mitotic activity and poor prognosis, indicating its role in leukemogenesis [32]. The results suggest that Subnetwork 5 may be related to metabolic reprogramming and stress response pathways. Altered metabolic states are known to support leukemic cell survival under therapeutic stress, and metabolic adaptation has been implicated as a key mechanism underlying resistance to targeted therapies [33,34].
- **Subnetwork 6**
  Subnetwork 6 includes *POU2AF1*, a transcriptional coactivator known to regulate B-cell differentiation and immune responses. Although primarily studied in lymphoid malignancies, recent transcriptomic analyses have revealed that *POU2AF1* is aberrantly expressed in AML, suggesting its potential involvement in leukemogenesis. Its expression correlates with deregulated immune signaling pathways and may reflect specific AML subtypes with immune-related gene expression profiles [35]. Subnetwork 6 may be associated with DNA damage response and repair mechanisms. Enhanced DNA repair capacity enables leukemic cells to tolerate therapeutic insults, thereby reducing treatment efficacy and contributing to the development of drug resistance [36,37].

**Table 3:** Identified biomarkers from Quizartinib resistant-specific gene network analysis. The biomarkers are categorized into subnetworks that they belong to. Their supporting evidence related to AML and Quizartinib is summarized.

Category	Gene	AML	Quizartinib
sub 1	IFIT1	Zhao et al. [13]	
	MX1		
	PSMB9	Yang et al. [14]	
	HLA-B	Hallner et al. [15]	
sub 2	GMFG	Zia et al. [17]	
	LAPTM5	Tsutsumi et al. [18]	
	ARHGDIB	Pont et al. [19]	
sub 3	NUPR1		
	GDF15	Lu and Liao [22]	Park et al. [23]
	TRIB3	Luo et al. [24]	
sub 4	UCHL1	Zhang et al. [27]	
	TSPYL5	Sawai et al. [28]	
	ID3	Zhao et al. [29]	
sub 5	RRM2	Cao et al. [31]	
	FAM64A	Archangelo et al. [32]	
sub 6	POU2AF1	Bolouri et al. [35]	

**Figure 3:**
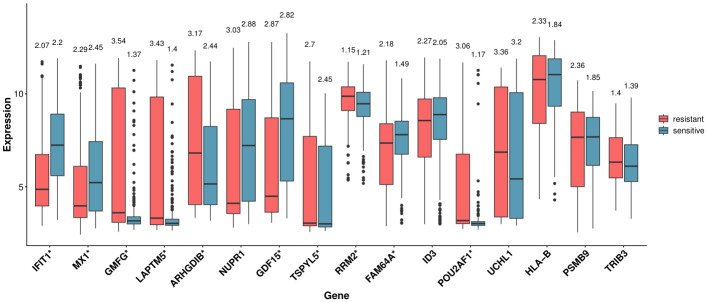
Expression levels of 16 hub genes in quizartinib-resistant and quizartinib-sensitive cell lines. In each boxplot, the central line represents the median, the box indicates the interquartile range (IQR), and the whiskers extend to 1.5 × IQR. Numbers displayed above each box represent the standard deviation (SD). Genes marked with an asterisk (*) on the *x*-axis indicate statistically significant differential expression between resistant and sensitive cell lines (p<0.05).

We further investigated whether these hub genes exhibited significant differences in expression according to Quizartinib sensitivity. The gene expression analysis shown in Figure 3 was performed using matched data from 892 cell lines that had both expression profiles and drug sensitivity (IC50) measurements, obtained by intersecting the original datasets (1020 cell lines for expression and 970 for drug sensitivity). Specifically, we selected the top 25% (n=223; most resistant) and bottom 25% (n=223; most sensitive) cell lines based on IC50 values for the statistical comparison. To this end, we compared the gene expression levels between cell lines with the top 25% IC50 values (resistant group) and those with the bottom 25% IC50 values (sensitive group), as shown in Figure 3. The corresponding gene expression data for the 16 genes across these selected cell lines are provided in the Supplementary Material. The observed outliers reflect inter-cell line variability rather than distinct cellular subpopulations, as each point corresponds to an independent cell line. In several genes, the distributions are asymmetric across phenotypes, with a subset of cell lines in one group exhibiting markedly higher or lower expression levels. This results in unidirectional outliers and suggests gene-specific heterogeneity associated with Quizartinib sensitivity. According to the Wilcoxon rank-sum test, 11 of the 16 identified hub genes exhibited significant differences in expression levels between the resistant and sensitive groups (*p* value < 0.05), and these genes are indicated with an asterisk (*) in the Figure 3.

**Figure 4:**
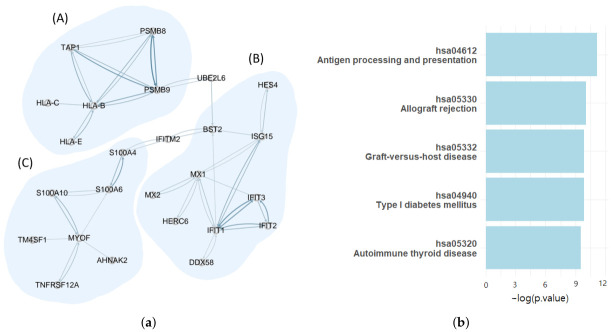
(**a**) Subnetwork 1 in Figure 2, visualized with three modules (A, B, and C). (**b**) Top five KEGG-enriched pathways identified from genes in Subnetwork 1.

Among the identified 6 Quizartinib-resistant-specific gene network, we focused on the largest subnetwork (i.e., subnetwork 1). As shown in Figure 4, Subnetwork 1 is visually divided into three modules (A, B, and C). Module A contains genes such as *HLA-B*, *HLA-C*, *HLA-E*, *PSMB8*, *PSMB9*, and *TAP1*, which are directly involved in MHC class I-mediated antigen processing and presentation. These genes are required for the presentation of intracellular peptides to cytotoxic T cells and are important for immune surveillance. Module B includes well-known interferon-stimulated genes (ISGs), such as *MX1*, *MX2*, *IFIT1–3*, *ISG15*, and *BST2*, which indicate antiviral immune responses. Module C consists of *S100A4*, *S100A6*, *S100A10*, *MYOF*, *AHNAK2*, *TM4SF1*, and *TNFRSF12A*, which are associated with cytoskeletal organization, membrane behavior, cell movement, features related to cancer invasiveness, and immune escape.

To validate the functional and structural integrity of the derived network, we first performed a pathway enrichment analysis on all genes in Subnetwork 1 using the Kyoto Encyclopedia of Genes and Genomes (KEGG). As summarized in Figure 4, the five most significantly enriched pathways were “Antigen processing and presentation” (hsa04612, *p* value = 1.40 × $10^{-5}$), “Allograft rejection” (hsa05330, *p* value = 4.37 × $10^{-5}$), “Graft-versus-host disease” (hsa05332, *p* value = 5.13 × $10^{-5}$), “Type I diabetes mellitus” (hsa04940, *p* value = 5.13 × $10^{-5}$), and “Autoimmune thyroid disease” (hsa05320, *p* value = 7.21 × $10^{-5}$). These immune-related pathways have also been reported to be significantly dysregulated in AML, particularly in transcriptome-wide analyses of AML patients with normal karyotype, in which multiple major histocompatibility complex (MHC) antigen presentation pathways—including antigen processing and presentation, allograft rejection, graft-versus-host disease, and autoimmune-related pathways—were consistently identified as significantly enriched and downregulated [38]. All the enriched pathways were related to immune function, suggesting that Subnetwork 1 was strongly linked to immune regulation. Among these pathways, “Antigen processing and presentation” was particularly important because they play key roles in presenting tumor antigens to cytotoxic T cells via MHC class I molecules. In AML, the reduced expression of genes such as *HLA-B* and *PSMB9* reportedly decreases immune recognition by T cells, thereby promoting immune escape and resistance to treatments, such as FLT3 inhibitors. Notably, pathway enrichment was mainly driven by genes from Module A, particularly *HLA-B*, *HLA-C*, and *TAP1*, whereas genes from Modules B and C contributed very little. These results indicate that MHC class I-related antigen presentation is a central component of the immune-related transcriptional pattern associated with Quizartinib resistance. Since KEGG pathway analysis considers only gene overlap and does not include gene–gene interactions, the strong agreement between the pathway enrichment results and the independently identified network modules supports the biological reliability of our network decomposition. Furthermore, to address the limitations of simple list-based enrichment and verify the network’s topological validity, we cross-referenced our co-expression edges with the STRING database. Remarkably, 46.43% of the identified edges were consistently supported by known protein-protein interactions or experimental evidence. Despite the inherent differences between transcript-level correlations and protein-level interactions, this substantial overlap with STRING and KEGG databases demonstrates that our network architecture accurately captures the functional and topological characteristics of the underlying biological system. Overall, these findings suggest that Subnetwork 1 captures immune-related transcriptional programs in Quizartinib-resistant AML, and that dysregulation of MHC class I-associated pathways may contribute to drug resistance and represent potential biomarkers or therapeutic targets.

We also performed Quizartinib sensitivity-specific gene network analysis using existing methods (i.e., DGNdetector, SAM-GS, GSCA). Since these methods cannot be directly applied to cell line-specific gene network inference, we first estimated gene networks for the top 10% of drug-sensitive and drug-resistant cell lines. Specifically, 178 cell lines with the lowest IC50 values were used to construct the drug-sensitive gene network, and 178 cell lines with the highest IC50 values were used for the drug-resistant gene network. We then applied the existing methods (i.e., DGNdetector, SAM-GS, GSCA) to identify drug resistance-specific gene networks. Table 4 shows the results of uncovering DGNs, where the columns “♯ of subnetworks (all)”, “♯ of subnetworks (5↑)”, and “Degree” represent the total number of identified DGNs, the number of DGNs containing more than 5 genes, and average node degree within identified DGNs, respectively.

As shown in Table 4, the existing methods (i.e., DGNdetector, SAM-GS, GSCA) detect a relatively larger number of DGNs compared to our strategy. In the simulation study, these methods were shown to be less effective in terms of false positives (TNR), suggesting that the larger number of DGNs they uncover may include truly significant DGNs. The complete set of DGNs identified by the existing methods (i.e., DGNdetector, SAM-GS, GSCA) is provided in the Supplementary Materials.

## 3. Discussion

In this study, we proposed a novel statistical framework for identifying phenotype-specific gene networks in a cell line-specific setting, addressing the key limitations of existing approaches. Traditional gene network inference methods typically construct a single population-level network, which overlooks regulatory heterogeneity among individual cell lines. However, each cell line can exhibit distinct gene–gene interactions due to the underlying molecular variability. Gene networks tend to be high-dimensional and complex, making their interpretation difficult. To address this challenge, we focused on identifying differentially regulated gene networks (DGNs) among phenotypes. Although several DGN methods have been developed to reveal condition-specific regulatory patterns, most are limited to population-level analyses and do not consider cell-line-level variations. Specifically, existing methods assess phenotypic specificity based only on between-group differences, which may not be sufficient for the accurate identification of phenotype-specific structures. Our method addresses this issue by evaluating both between-phenotype and within-phenotype heterogeneity. By applying this criterion, we can effectively identify networks that are not only distinct across phenotypes, but also consistently observed within each phenotype.

Simulation studies demonstrated that the proposed strategy outperformed existing approaches in detecting DGN in a cell-line-specific scenario. We applied our method to the GDSC dataset to investigate the gene networks associated with resistance to Quizartinib, an FDA-approved drug for AML. Our analysis revealed immune-related networks that were specifically enriched in Quizartinib-resistant cell lines. In particular, hub genes involved in MHC class I-mediated antigen presentation have emerged as key components of resistance-related subnetworks. These findings suggest that immune evasion mechanisms play a significant role in drug resistance and highlight potential targets for therapeutic interventions.

Taken together, our method provides a powerful and interpretable approach for the detection of phenotype-specific molecular interactions in a cell line-specific context. Beyond AML, this strategy may be broadly applicable to other diseases and drug response studies where cell line-level heterogeneity is important.

In differentially regulated gene network identification, accurate network estimation is crucial, as errors can propagate and affect downstream analyses of cell line-specific interactions. Tuning parameters in kernel-based $L_{1}$ regularization for precision matrix estimation strongly influence network structure and, consequently, DGNs identification performance; overly stringent or lenient choices may produce networks that are too sparse or too dense, increasing false negatives or positives. Although network estimation based on tuning parameters was beyond the scope of this study, we focused on uncovering DGNs using pre-estimated networks while acknowledging that tuning parameter choices can affect downstream DGN identification. Supplementary Materials presents the results of DGNs obtained using fixed extreme values of the tuning parameters, highlighting the importance of careful network estimation for reliable downstream analyses.

We acknowledge that our study focuses on the development of a statistical framework for comparing phenotype-specific gene regulatory networks within a cell line-specific context and does not explicitly incorporate network clustering or module detection. This represents a limitation, as the biological interpretability of differential network patterns may depend on the quality of upstream network construction and the identification of meaningful modular structures. In particular, incorporating well-defined clustering or module detection approaches could facilitate a more systematic understanding of coordinated gene interactions across phenotypic conditions. Future work will focus on integrating statistically rigorous network clustering or module detection methods to further enhance the interpretability and practical utility of the proposed framework.

Although our strategy provides a systematic approach for identifying phenotype-specific gene regulatory networks in the cell line specific framework, this study is primarily based on statistical and bioinformatic analyses and does not include experimental validation. Although several of our findings are supported by the existing literature, we acknowledge that such indirect evidence cannot fully substitute for direct biological confirmation. Thus, the identified subnetworks and candidate biomarkers should be interpreted as hypothesis-generating results that require further validation through experimental studies. Future work will focus on validating these findings using in vitro or in vivo experiments and extending the framework to incorporate additional biological data sources.

Comparative analyses show that existing DGN methods often focus on population-level networks and only consider between-group differences, missing cell line-specific heterogeneity. In contrast, our method evaluates both within-phenotype and between-phenotype variability, enabling more accurate detection of DGNs (see Table 1 and Table 2).

Furthermore, immune-related subnetworks identified in Quizartinib-resistant AML cell lines are consistent with prior studies highlighting MHC class I-mediated antigen presentation and immune evasion in drug resistance. This supports the biological relevance of our findings and demonstrates how the proposed framework improves upon existing approaches by integrating statistical rigor with interpretable biological insights.

One limitation of this study is that genomic characteristics of the cell lines, such as mutation or genotype profiles, were not explicitly incorporated into the analysis. Given that such genomic features are known to play a critical role in drug resistance mechanisms and may have prognostic value, their inclusion could further enhance the biological interpretability and predictive capability of the proposed framework. Future work will focus on integrating multi-omics data, including genomic alterations, to provide a more comprehensive understanding of phenotype-associated network dynamics.

## 4. Method

### 4.1. Previous Methodologies for Identifying Differential Molecular Interplays

Existing methodologies for identifying differentially regulated gene networks (DGNs) traditionally follow two main analytical directions: (1) differential expression [10] and (2) differential co-expression [11] studies.

#### 4.1.1. Differential Expression Studies

Existing analyses focus on identifying genes or gene sets whose mean expression levels change between phenotypes, such as cancer and normal. Genes or gene sets whose mean expression levels either increase or decrease are generally considered to be associated with the phenotype. Let $X\in R^{n_{A}\times p}$ and $Y\in R^{n_{B}\times p}$ denote the expression levels of *p* genes for the phenotypes A and B, respectively. SAM-GS [10] measures differences in expression levels between phenotypes as follows:

(7) $$D_{\text{SAM-GS}}=\sum_{j=1}^{p}\frac{{\left({\bar{x}}_{j}-{\bar{y}}_{j}\right)}^{2}}{s_{j}+s_{0}},$$

where ${\bar{x}}_{j}$ and ${\bar{y}}_{j}$ are the averages of the expression levels of the *j*th gene in phenotypes A and B, respectively; $s_{0}$ is a small positive constant for variance stabilization; and $s_{j}$ is a gene-specific scatter defined as

(8) $$s_{j}=\sqrt{a\left\{\sum_{i=1}^{n_{A}}{\left(x_{ij}-{\bar{x}}_{j}\right)}^{2}+\sum_{i=1}^{n_{B}}{\left(y_{ij}-{\bar{y}}_{j}\right)}^{2}\right\}},\;\mathrm{where}\;a=\frac{1/n_{A}+1/n_{B}}{n_{A}+n_{B}-2}.$$

A large value of $D_{\text{SAM-GS}}$ indicates that the corresponding gene set shows differences in mean expression between phenotypes.

#### 4.1.2. Differential Co-Expression Studies

Differential co-expression studies have focused on identifying gene sets whose co-expression patterns vary between phenotypes. In this approach, differences in co-expression patterns were assumed to reflect changes in regulatory mechanisms. GSCA [11] summarizes the differences in pairwise correlations within a given gene as follows:

(9) DGSCA=1nk∑k≠j∑j=1p(cjkA−cjkB)2,

where $c_{jk}^{A}$ and $c_{jk}^{B}$ denote the Pearson’s correlation coefficients between *j*th and *k*th genes for phenotypes A and B, respectively. If the value of $D_{\mathrm{GSCA}}$ is large, then the overall co-expression patterns of the gene sets differ significantly between phenotypes. The two gene set analysis methods (i.e., SAM-GS and GSCA) can be applied to differential gene network analysis, in which the node set of the gene network *V* is considered a gene set.

Although these methods have often been used to uncover phenotype-associated gene sets and networks, existing methods (i.e., DGNdetector, SAM-GS, GSCA) are based on incomplete information (i.e., expression levels of genes and their correlation). In real biological contexts, disease phenotypes are not driven by shifts in mean expression levels or co-expression patterns alone; rather, the underlying gene–gene regulatory interactions play a crucial role. Consequently, these methods often fail to identify the disease-related molecular interactions.

#### 4.1.3. DGNdetector

To address these limitations, a DGNdetector was proposed that incorporates both gene expression levels and the network structure, specifically defined by the presence of edges, their functional weights, and the underlying sparsity pattern of the precision matrix ($\Omega =\Sigma^{-1}$), which encodes the conditional dependencies between genes as described in Equation (10) [7]. This method assumes that the gene expression vectors of the two phenotypes follow a multivariate normal distribution.

(10) $$x∼N_{p}\left(\mu_{A},\Sigma_{A}\right)\;\mathrm{and}\;y∼N_{p}\left(\mu_{B},\Sigma_{B}\right),$$

where $\mu_{A}$ and $\mu_{B}$ denote the mean expression profiles for phenotype A and B, respectively, and $\Sigma_{A}$ and $\Sigma_{B}$ denote their corresponding p×p covariance matrices. Under the assumptions in (10), the probability density functions of x and y are given by:

(11) $$\begin{array}{cc} f(x|\mu_{A},\Sigma_{A}) & ={\left(2\pi \right)}^{-\frac{p}{2}}{\left|\Sigma_{A}\right|}^{-\frac{1}{2}}\exp\left(-\frac{1}{2}{\left(x-\mu_{A}\right)}^{⊤}\Sigma_{A}^{-1}\left(x-\mu_{A}\right)\right), \end{array}$$

(12) $$\begin{array}{cc} g(y|\mu_{B},\Sigma_{B}) & ={\left(2\pi \right)}^{-\frac{p}{2}}{\left|\Sigma_{B}\right|}^{-\frac{1}{2}}\exp\left(-\frac{1}{2}{\left(y-\mu_{B}\right)}^{⊤}\Sigma_{B}^{-1}\left(y-\mu_{B}\right)\right). \end{array}$$

The DGNdetector measures the difference of the networks based on the closeness between probability density function $f(x|\mu_{A},\Sigma_{A})$ and $g(y|\mu_{B},\Sigma_{B})$, which enable us to utilize richer information to identify DGNs. To quantify this difference, this method employs the KL-divergence, which is a measure of how one distribution diverges from another. In the case of two multivariate normal distributions, the KL divergence is given as follows:

(13) $$\mathrm{KL}\left(f\|g\right)=\int_{-\infty }^{\infty }f\left(x\right)\log\left\{\frac{f(x)}{g(x)}\right\}dx.$$

The statistics are summarized as follows:

(14) $$D_{\mathrm{KL}}=\frac{1}{2}\left[\log\left\{\frac{|\Sigma_{B}|}{|\Sigma_{A}|}\right\}-p+\mathrm{tr}\left\{\Sigma_{B}^{-1}\Sigma_{A}\right\}+{\left(\mu_{A}-\mu_{B}\right)}^{⊤}\Sigma_{B}^{-1}\left(\mu_{A}-\mu_{B}\right)\right],$$

where $\Sigma_{A}$ and $\Sigma_{B}$ are obtained from the corresponding precision matrices that encode the underlying network structure through conditional dependencies among genes. By incorporating both gene expression levels and network structures, the DGNdetector provides more comprehensive information for identifying DGNs.

However, the existing approaches have focused primarily on between-phenotype heterogeneity. These methods typically assume that each phenotype can be represented by a single representative network and detect differential regulation by evaluating the differences between these phenotype-level networks. However, this representative network assumption ignores the variations that exist within each phenotype. In other words, conventional frameworks perform phenotype-level comparisons but do not explicitly evaluate within-phenotype homogeneity, which is essential for differentially regulated cell line-specific gene network analysis. As a result, the existing methods (i.e., DGNdetector, SAM-GS, GSCA) are not well suited for cell line-specific gene network analysis.

To identify phenotype-specific characteristics in cell line-specific gene network analysis, two essential conditions should be considered: (1) the query network must differ from networks of other phenotypes (between-phenotype heterogeneity), and (2) the query network must be consistently observed among cell lines within the same phenotype (within-phenotype homogeneity). These two complementary conditions jointly define phenotype-specific characteristics.

### 4.2. Proposed Strategy

We propose a novel statistical framework that simultaneously evaluates the between-phenotype heterogeneity and within-phenotype homogeneity to identify differentially regulated cell line-specific gene networks.

**Figure 5:**
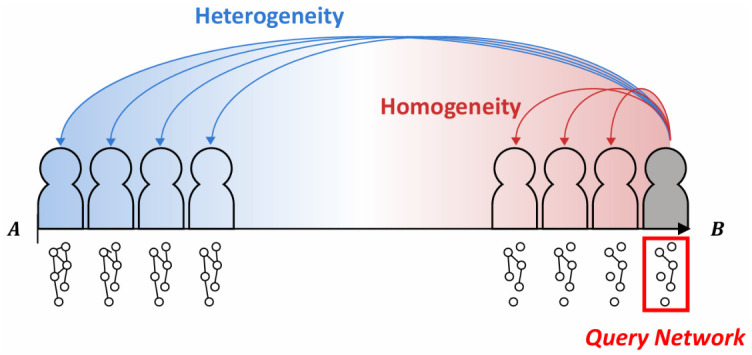
A schematic of the cell line-specific setting. Cell lines are ordered by a modulator (e.g., drug sensitivity) and split into phenotypes A and B. The red box marks the query cell line $i^{*}\in B$, which serves as the reference network for comparison. Blue arrows represent between-phenotype variations from $i^{*}$ to cell lines in A (heterogeneity), and red arrows represent within-phenotype variations from $i^{*}$ to cell lines in B (homogeneity). The grey region highlights the query network associated with $i^{*}$ and does not indicate an intermediate class or filtered samples; no additional cutoff or filtering is applied in this schematic.

Let A and B denote the sets of cell lines belonging to phenotypes A and B, respectively, and $n_{A}$ and $n_{B}$ denote cell line sizes. We considered a query cell line $i^{*}\in B$, highlighted by the red box in Figure 5 to evaluate how well the network of the $i^{*}$ cell line reflects phenotype B-specificity. For each cell line *i*, we summarize its cell line-specific gene network using a Gaussian distribution $N({\hat{\mu }}_{i},{\hat{\Sigma }}_{i})$ where ${\hat{\mu }}_{i}$ is the observed gene expression vector of cell line *i* and ${\hat{\Sigma }}_{i}$ is the covariance matrix obtained from the cell line-specific precision matrix (i.e., the edge structure). The closeness between two cell line-specific networks was quantified by KL-divergence, providing a cell-line-level measure of network differences.

We define between-phenotype heterogeneity as the average KL divergence from the query network of cell line $i^{*}\in B$ to the networks in the other phenotype A, as follows:

(15) $$D_{\mathrm{between}}\left(i^{*}\right)=\frac{1}{n_{A}}\sum_{i\in A}\mathrm{KL}\left(i^{*},i\right),$$

where $\mathrm{KL}(i^{*},i)$ denotes the KL divergence between the gene networks of $i^{*}$th and *i*th cell lines. The large value of $D_{\mathrm{between}}\left(i^{*}\right)$ indicates that the network of the $i^{*}$th cell line is highly heterogeneous compared to that of phenotype A. We also define within-phenotype homogeneity as the average KL divergence of the query network of cell line $i^{*}\in B$ from the networks in the same phenotype B as follows:

(16) $$D_{\mathrm{within}}\left(i^{*}\right)=\frac{1}{n_{B}}\sum_{j\in B}\mathrm{KL}\left(i^{*},j\right).$$

The large value of $D_{\mathrm{within}}\left(i^{*}\right)$ indicates that the network of the $i^{*}$th cell line is highly heterogeneous compared to that of phenotype B. To identify DGNs based on between-phenotype heterogeneity and within-phenotype homogeneity, we proposed the following statistics:

(17) $$D_{\text{KL-ratio}}\left(i^{*}\right)=\frac{D_{\mathrm{between}}\left(i^{*}\right)}{D_{\mathrm{within}}\left(i^{*}\right)}.$$

The numerator measures between-phenotype divergence, whereas the denominator measures within-phenotype divergence. Hence, a larger value of $D_{\text{KL-ratio}}\left(i^{*}\right)$ indicates higher between-phenotype variation and lower within-phenotype variation. Therefore, larger statistic values indicate that the $i^{*}$th query network is more likely to be phenotype-specific.

**Figure 6:**
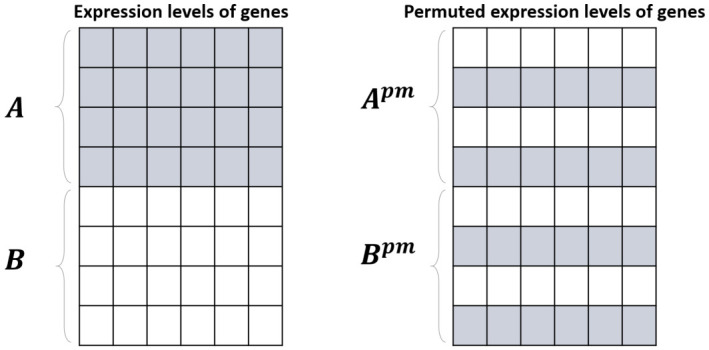
The permutation framework. The permuted cell lines for two phenotypes are generated by randomly shuffling their labels for each permutation.

To assess the significance of $D_{\text{KL-ratio}}\left(i^{*}\right)$, we consider a phenotype-based permutation framework. Here, the null hypothesis states that all cell lines have random network structures, meaning that the network structure observed in each cell line arises randomly and is independent of cell line characteristics. In contrast, the alternative hypothesis states that cell lines with extreme values of characteristics have cell line-specific, non-random network structures. The observed $D_{\text{KL-ratio}}\left(i^{*}\right)$, which is significantly larger than the values from the permutation-based null distribution, provides evidence that the network structure of the query cell line $i^{*}$ exhibits a cell line-specific pattern that cannot be explained by random variation alone. For each permutation, pm=1,⋯,T, this produces a new labeling of the same cell lines, which we denote as the permuted datasets $A^{\mathrm{pm}}$ and $B^{\mathrm{pm}}$, as shown in Figure 6. We then compute the statistics for each permuted dataset as follows:

(18) $$D_{\text{KL-ratio}}^{\mathrm{pm}}\left(i^{*}\right)=\frac{\frac{1}{n_{A}}\sum_{i\in A^{\mathrm{pm}}}\mathrm{KL}\left(i^{*},i\right)}{\frac{1}{n_{B}}\sum_{j\in B^{\mathrm{pm}}}\mathrm{KL}\left(i^{*},j\right)}.$$

The *p*-value was obtained as follows:

(19) $$p.\mathrm{value}=\frac{\sum_{\mathrm{pm}=1}^{T}I\left(D_{\text{KL-ratio}}\leq D_{\text{KL-ratio}}^{\mathrm{pm}}\right)}{T},$$

where I(·) is the indicator function.

The proposed strategy considers how different the networks are between phenotypes and how similar they are within each phenotype. A large statistical value in (18) indicates that the network differs significantly between phenotypes and remains consistent within the phenotype, suggesting that the network is truly phenotype-specific. Our framework captures both heterogeneity (i.e., differences between phenotypes) and homogeneity (i.e., similarities within phenotypes). This allows for a more complete understanding of how gene networks change between phenotypes in cell line-specific or cell line-level network analysis. This provides a statistically reliable method of determining whether a network pattern is truly specific to a particular phenotype.

## Figures and Tables

**Table 1:** Differentially regulated gene network identification result 1; p=100 genes, where NaN indicates that no subnetwork was selected (TPR = 0); therefore, precision is zero and the F-measure was not computed. Network topologies: random (RD), scale-free (SF), hub (HUB), cluster (CL), and band (BD).

		Scenario 1 (0/0)					Scenario 2 (0/1)					Scenario 3 (0/5)				
		RD	SF	HUB	CL	BD	RD	SF	HUB	CL	BD	RD	SF	HUB	CL	BD
Accuracy	CellDGN	0.766	0.764	**0.746**	0.792	**0.845**	**0.996**	**0.996**	**1.000**	**0.996**	**1.000**	**0.994**	**0.992**	**0.996**	**1.000**	**1.000**
	DGNdetector	0.742	0.706	0.734	0.720	0.770	0.918	0.932	0.940	0.946	0.875	0.924	0.926	0.934	0.940	0.950
	SAM-GS	0.468	0.482	0.492	0.476	0.470	0.916	0.904	0.910	0.904	0.915	0.962	0.976	0.956	0.970	0.985
	GSCA	0.450	0.436	0.410	0.462	0.410	0.500	0.458	0.424	0.490	0.420	0.640	0.694	0.608	0.714	0.530
	DiffNetFDR	**0.798**	**0.794**	0.718	**0.860**	0.840	0.824	0.748	0.812	0.826	0.795	0.858	0.824	0.782	0.868	0.725
Precision	CellDGN	**0.993**	**1.000**	**0.992**	**1.000**	**0.986**	**0.992**	**0.996**	**1.000**	**0.992**	**1.000**	**0.988**	**0.984**	**0.992**	**1.000**	**1.000**
	DGNdetector	0.810	0.770	0.842	0.820	0.807	0.859	0.880	0.893	0.903	0.800	0.868	0.871	0.883	0.893	0.909
	SAM-GS	0.136	0.154	0.300	0.125	0.125	0.909	0.947	0.952	0.939	0.928	0.933	0.954	0.926	0.943	0.971
	GSCA	0.281	0.204	0.021	0.349	0.000	0.500	0.338	0.104	0.459	0.056	0.713	0.771	0.729	0.786	0.579
	DiffNetFDR	0.761	0.741	0.660	0.838	0.798	0.787	0.690	0.755	0.788	0.766	0.830	0.787	0.724	0.854	0.703
TPR	CellDGN	0.536	0.528	0.496	**0.584**	0.700	**1.000**	0.996	**1.000**	**1.000**	**1.000**	**1.000**	**1.000**	**1.000**	**1.000**	**1.000**
	DGNdetector	0.632	0.588	0.576	0.564	0.710	**1.000**	**1.000**	**1.000**	**1.000**	**1.000**	**1.000**	**1.000**	**1.000**	**1.000**	**1.000**
	SAM-GS	0.012	0.008	0.012	0.008	0.010	0.924	0.856	0.864	0.864	0.900	0.996	**1.000**	0.992	**1.000**	**1.000**
	GSCA	0.064	0.044	0.004	0.088	0.000	0.164	0.088	0.020	0.112	0.010	0.468	0.552	0.344	0.588	0.220
	DiffNetFDR	**0.868**	**0.904**	**0.900**	**0.892**	**0.910**	0.888	0.900	0.924	0.892	0.850	0.900	0.888	0.912	0.888	0.780
TNR	CellDGN	**0.996**	**1.000**	**0.996**	**1.000**	**0.990**	**0.992**	**0.996**	**1.000**	**0.992**	**1.000**	**0.988**	**0.984**	**0.992**	**1.000**	**1.000**
	DGNdetector	0.852	0.824	0.892	0.876	0.830	0.836	0.864	0.892	0.892	0.750	0.868	0.852	0.880	0.880	0.900
	SAM-GS	0.924	0.956	0.972	0.944	0.930	0.908	0.952	0.956	0.944	0.930	0.920	0.952	0.920	0.940	0.970
	GSCA	0.836	0.828	0.816	0.836	0.820	0.836	0.828	0.816	0.868	0.830	0.812	0.836	0.872	0.840	0.840
	DiffNetFDR	0.728	0.684	0.536	0.828	0.770	0.760	0.596	0.700	0.760	0.740	0.816	0.760	0.652	0.848	0.670
F-measure	CellDGN	0.696	0.691	0.661	0.737	0.818	**0.996**	**0.996**	**1.000**	**0.996**	**1.000**	**0.994**	**0.992**	**0.996**	**1.000**	**1.000**
	DGNdetector	0.710	0.667	0.684	0.668	0.755	0.924	0.936	0.943	0.949	0.889	0.929	0.931	0.938	0.943	0.952
	SAM-GS	0.022	0.015	0.023	0.015	0.019	0.917	0.899	0.906	0.900	0.914	0.963	0.977	0.958	0.971	0.985
	GSCA	0.104	0.072	0.007	0.141	Nan	0.247	0.140	0.034	0.180	0.017	0.565	0.643	0.467	0.673	0.319
	DiffNetFDR	**0.811**	**0.814**	**0.761**	**0.864**	**0.850**	0.835	0.781	0.831	0.837	0.806	0.864	0.835	0.807	0.871	0.739

**Table 2:** Differentially regulated gene network identification result 1; p=200 genes, where NaN indicates that no subnetwork was selected (TPR = 0); therefore, precision is zero and the F-measure was not computed. Network topologies: random (RD), scale-free (SF), hub (HUB), cluster (CL), and band (BD).

		Scenario 1 (0/0)					Scenario 2 (0/1)					Scenario 3 (0/5)				
		RD	SF	HUB	CL	BD	RD	SF	HUB	CL	BD	RD	SF	HUB	CL	BD
Accuracy	CellDGN	0.802	**0.826**	0.800	0.810	**0.880**	**1.000**	**1.000**	**1.000**	**1.000**	**1.000**	**0.998**	**0.996**	**1.000**	**1.000**	**1.000**
	DGNdetector	0.638	0.676	0.638	0.640	0.750	0.934	0.944	0.916	0.950	0.935	0.938	0.914	0.940	0.958	0.930
	SAM-GS	0.460	0.474	0.484	0.488	0.475	0.928	0.932	0.942	0.946	0.940	0.984	0.968	0.986	0.972	0.985
	GSCA	0.326	0.352	0.354	0.324	0.325	0.372	0.310	0.362	0.372	0.345	0.604	0.526	0.564	0.626	0.520
	DiffNetFDR	**0.848**	0.810	**0.852**	**0.824**	0.805	0.832	0.796	0.860	0.828	0.805	0.882	0.832	0.900	0.828	0.790
Precision	CellDGN	**0.981**	**1.000**	**1.000**	**1.000**	**1.000**	**1.000**	**1.000**	**0.996**	**1.000**	**1.000**	**0.996**	**0.992**	**1.000**	**1.000**	**1.000**
	DGNdetector	0.752	0.814	0.732	0.787	0.868	0.883	0.899	0.859	0.912	0.885	0.890	0.853	0.893	0.923	0.877
	SAM-GS	0.000	0.000	0.167	0.200	0.000	0.921	0.954	0.959	0.941	0.949	0.969	0.940	0.973	0.947	0.971
	GSCA	0.000	0.013	0.000	0.011	0.000	0.100	0.039	0.084	0.056	0.000	0.631	0.533	0.586	0.644	0.526
	DiffNetFDR	0.822	0.788	0.828	0.796	0.765	0.812	0.770	0.844	0.806	0.752	0.872	0.796	0.910	0.799	0.734
TPR	CellDGN	0.616	0.652	0.600	0.620	0.760	**1.000**	**1.000**	**1.000**	**1.000**	**1.000**	**1.000**	**1.000**	**1.000**	**1.000**	**1.000**
	DGNdetector	0.412	0.456	0.436	0.384	0.590	**1.000**	**1.000**	0.996	0.996	**1.000**	**1.000**	**1.000**	**1.000**	**1.000**	1.000
	SAM-GS	0.000	0.000	0.008	0.008	0.000	0.936	0.908	0.924	0.952	0.930	**1.000**	**1.000**	**1.000**	**1.000**	**1.000**
	GSCA	0.000	0.004	0.000	0.004	0.000	0.032	0.016	0.028	0.016	0.000	0.500	0.424	0.436	0.564	0.400
	DiffNetFDR	**0.888**	**0.848**	**0.888**	**0.872**	**0.880**	0.864	0.844	0.884	0.864	0.910	0.896	0.892	0.888	0.876	0.910
TNR	CellDGN	**0.988**	**1.000**	**1.000**	**1.000**	**1.000**	**1.000**	**1.000**	**0.992**	**1.000**	**0.996**	**0.996**	**0.992**	**1.000**	**1.000**	**1.000**
	DGNdetector	0.864	0.896	0.840	0.896	0.910	0.868	0.888	0.836	0.904	0.870	0.876	0.828	0.880	0.916	0.860
	SAM-GS	0.920	0.948	0.960	0.968	0.950	0.920	0.956	0.960	0.940	0.950	0.968	0.936	0.972	0.944	0.970
	GSCA	0.652	0.700	0.708	0.644	0.650	0.712	0.604	0.696	0.728	0.690	0.708	0.628	0.692	0.688	0.640
	DiffNetFDR	0.808	0.772	0.816	0.776	0.730	0.800	0.748	0.836	0.792	0.700	0.868	0.772	0.912	0.780	0.670
F-measure	CellDGN	0.757	0.789	0.750	0.765	**1.000**	**1.000**	**1.000**	**0.996**	**1.000**	**1.000**	**0.998**	**0.996**	**1.000**	**1.000**	**1.000**
	DGNdetector	0.532	0.585	0.546	0.516	0.702	0.938	0.947	0.922	0.952	0.939	0.942	0.921	0.943	0.960	0.935
	SAM-GS	0.000	0.000	0.015	0.015	Nan	0.929	0.930	0.941	0.946	0.939	0.984	0.969	0.986	0.973	0.985
	GSCA	0.000	0.006	0.000	0.006	Nan	0.048	0.023	0.042	0.025	Nan	0.558	0.472	0.500	0.601	0.455
	DiffNetFDR	**0.854**	**0.817**	**0.857**	**0.832**	0.819	0.837	0.805	0.863	0.834	0.824	0.884	0.842	0.899	0.836	0.812

**Table 4:** Differentially regulated gene network identification results by using existing methods (i.e., DGNdetector, SAM-GS, GSCA).

Methods	♯ Subnetworks (All)	♯ Subnetworks (5↑)	Degree
Our strategy	25	6	7.2
DGNdetector	45	9	9.5
SAM-GS	59	11	8.3
GCSC	21	9	18.4
DiffNetFDR	10	0	1.0

## Data Availability

The datasets used in the AML drug sensitivity-specific gene network analysis are available from the Genomics of Drug Sensitivity in Cancer (GDSC) database (www.cancerrxgene.org) accessed on 1 March 2025. The code used in this study is publicly available on GitHub at: https://github.com/JooeeOh/ssDGN. The repository includes the implementation of the proposed method along with instructions for reproducing the analysis.

## Supplementary Materials

The following supporting information can be downloaded at: https://www.mdpi.com/article/10.3390/ijms27093885/s1.

## Glossary

Glossary of mathematical symbols used in the manuscript:

**Symbol**	**Definition**
*p*	Number of genes (dimension of expression vector)
*n*	Total number of cell lines
α	Index for cell lines (α=1,…,n)
*ℓ*	Index for target gene in nodewise regression
A,B	Sets of cell lines belonging to phenotype A and B
$n_{A},n_{B}$	Number of cell lines in phenotype A and B
nw	Subnetwork index (nw=1,…,10 in simulations)
Ω	Baseline precision matrix (inverse covariance matrix)
$\Omega_{\alpha }$	Cell line-specific precision matrix for the αth cell line
${\hat{\Omega }}_{\alpha }$	Estimated cell line-specific precision matrix
Σ	Covariance matrix
$\Sigma_{\alpha }$	Covariance matrix of the αth cell line
${\hat{\Sigma }}_{\alpha }$	Estimated covariance matrix obtained from ${\hat{\Omega }}_{\alpha }^{-1}$
$v_{\alpha }$	Cell line-specific scaling factor for precision matrix generating
$m_{\alpha }$	Modulator value for cell line α
$\mu_{A},\mu_{B}$	Phenotype-specific mean expression vectors
${\hat{\mu }}_{\alpha }$	Observed gene expression vector for the αth cell line
$x_{\alpha }^{\left(nw\right)}$	Expression levels of genes in the nwth subnetwork for the αth cell line
${\hat{\beta }}_{\ell \alpha }$	Estimated edge weigth vector for the *ℓ*th target gene in the αth cell line
$D_{KL}$	Kullback–Leibler divergence between two networks
$D_{\mathrm{between}}\left(i^{*}\right)$	Between-phenotype heterogeneity measure for query cell line $i^{*}$
$D_{\mathrm{within}}\left(i^{*}\right)$	Within-phenotype homogeneity measure for query cell line $i^{*}$

## Appendix A. Derive the Precision Matrix

We first introduce notation for block submatrices of the covariance matrix and then connect the block inverse representation of the precision matrix to a population linear regression. Let Σ be a p×p positive definite covariance matrix. For a fixed index ℓ∈1,⋯,p, and let $\Sigma_{-\ell ,-\ell }$ denote the (p−1)×(p−1) submatrix obtained by removing *ℓ*th row and column of Σ. Similarily. Let $\Sigma_{\ell ,-\ell }$ denote the *ℓ*th row of Σ with its *ℓ*th element removed, and let $\Sigma_{-\ell ,\ell }$ denote the *ℓ*th column of Σ with its *ℓ*th element removed. By Schur complement, the *ℓ*th diagonal element of Ω is

(A1) $$\Omega_{\ell ,\ell }={\left(\Sigma_{\ell ,\ell }-\Sigma_{\ell ,-\ell }\Sigma_{-\ell ,-\ell }^{-1}\Sigma_{-\ell ,\ell }\right)}^{-1},$$

and the *ℓ*th row of Ω with the *ℓ*th element removed is

(A2) $$\Omega_{\ell ,-\ell }=-{\left(\Sigma_{\ell ,\ell }-\Sigma_{\ell ,-l}\Sigma_{-\ell ,-\ell }^{-1}\Sigma_{-\ell ,\ell }\right)}^{-1}\Sigma_{\ell ,-l}\Sigma_{-\ell ,-\ell }^{-1}=-\Omega_{\ell ,\ell }\Sigma_{\ell ,-\ell }\Sigma_{-\ell ,-\ell }^{-1}.$$

We now relate (A1) and (A2) to a linear regression. Let $r_{t,\ell }^{*}:=r_{t,\ell }-\frac{1}{n}\sum_{t=1}^{n}r_{t,\ell }$ be the vector collecting all centered variables except the *ℓ*th one. Define $\beta_{\ell }$ as the vector β of length p−1 that minimizes $E{\left[r_{t,\ell }^{*}-{\left(r_{t,-\ell }^{*}\right)}^{⊤}\beta \right]}^{2}$. We get the solution

(A3) $$\beta_{\ell }=\Sigma_{-\ell ,-\ell }^{-1}\Sigma_{-\ell ,\ell }.$$

Using (A3), we can rewrite (A2) as

(A4) $$\Omega_{\ell ,-\ell }=-\Omega_{\ell ,\ell }\beta_{\ell }^{⊤}.$$

Define the regression residual $\eta_{t,\ell }:=r_{t,\ell }^{*}-{\left(r_{t,-\ell }^{*}\right)}^{⊤}\beta_{\ell }$. By (A3), we can verify that

(A5) $$\begin{array}{cc} E[r_{t,\ell }^{*}\eta_{t,\ell }] & =E\left[r_{t,\ell }^{*}r_{t,\ell }^{*}\right]-E\left[r_{t,\ell }^{*}{\left(r_{t,-\ell }^{*}\right)}^{⊤}\right]\beta_{\ell }\\ \; & =\Sigma_{-\ell ,\ell }-\Sigma_{-\ell ,-\ell }\Sigma_{-\ell ,-\ell }^{-1}\Sigma_{-\ell ,\ell }=0. \end{array}$$

And, we can write the population regression model

(A6) $$r_{t,\ell }^{*}={\left(r_{t,-\ell }^{*}\right)}^{⊤}\beta_{\ell }+\eta_{t,\ell }.$$

We now use the result above to derive a formula for Ω. Using (A3), (A5) and (A6), we get

(A7) $$\begin{array}{cc} \Sigma_{\ell ,\ell }=E{\left[r_{t,\ell }^{*}\right]}^{2} & =\beta_{\ell }^{⊤}\Sigma_{-\ell ,-\ell }\beta_{\ell }+E\left[\eta_{t,l\ell }^{2}\right]\\ \; & =\Sigma_{\ell ,-\ell }\Sigma_{-\ell ,-\ell }^{-1}\Sigma_{-\ell ,\ell }+E{\left[\eta_{t,l}\right]}^{2}. \end{array}$$

Define $\sigma_{\ell }^{2}:=E\left[\eta_{t,\ell }^{2}\right]$. Then

(A8) $$\sigma_{\ell }^{2}=\Sigma_{\ell ,\ell }-\Sigma_{-\ell ,-\ell }\Sigma_{-\ell ,-\ell }^{-1}\Sigma_{-\ell ,\ell }=\frac{1}{\Omega_{\ell ,\ell }},$$

Comparing this expression with the Schur complement term in the block inverse formula yields

(A9) $$\Omega_{\ell ,\ell }=\frac{1}{\sigma_{\ell }^{2}}.$$

Finally, combining the results gives

(A10) $$\Omega_{\ell ,-\ell }=-\Omega_{\ell ,\ell }\beta_{\ell }^{⊤}=-\frac{\beta_{\ell }^{⊤}}{\sigma_{\ell }^{2}}$$
